# Gastric and duodenal squamous cell carcinoma: metastatic or primary?

**DOI:** 10.1186/1477-7819-11-204

**Published:** 2013-08-19

**Authors:** Jian-bin Hu, Yan-hong Zhu, Mei Jin, Xiao-nan Sun

**Affiliations:** 1 Department of Radiation Oncology, Sir Run Run Shaw Hospital of Zhejiang University School of Medicine, Hangzhou, Zhejiang Province 310016, China; 2 Department of Pathology, Sir Run Run Shaw Hospital of Zhejiang University School of Medicine, Hangzhou, Zhejiang Province 310016, China

**Keywords:** Gastrointestinal tract, Lung cancer, Metastasis, Squamous cell carcinoma

## Abstract

Either metastatic or primary squamous cell carcinoma in the gastrointestinal tract is extremely rare, with very few cases reported in the literature. In this paper, we report a case in which the patient presented with dysphagia during the course of radiotherapy for recurrent lung cancer in a mediastinal lymph node. Although the dysphagia mimicked radiation esophagitis, the ultimate cause proved to be gastric and duodenal metastases from primary lung squamous cell carcinoma. Taking into account the value of identification of metastatic or primary SCC in the stomach and duodenum on the prognosis and treatment options, it is imperative that the correct diagnosis be established. This report is followed by a discussion of the differential diagnosis between metastatic and primary squamous cell carcinoma in the stomach and duodenum.

## Background

Either metastatic or primary squamous cell carcinoma (SCC) in the gastrointestinal tract is extremely rare, with very few cases reported in the literature. The gastrointestinal tract has rarely been reported as a metastatic site of various tumors, such as lung cancer, breast cancer, hepatocellular carcinoma, melanoma, testicular seminoma, choriocarcinoma, Merkel cell carcinoma, malignant fibrous histiocytoma and others [1-4]. Most occurrences of metastatic SCC involving the gastrointestinal tract originate from lung primary tumors, but these occurrences remain extremely rare, with very few cases of stomach or duodenal involvement reported in the literature [5,6].

The incidence of primary SCC of the stomach or duodenum is also very low. It is estimated that the worldwide incidence of primary SCC of the stomach is 0.04% to 0.07% [7,8]. Fewer than ten cases of primary SCC of the duodenum have been noted [9]. In this report, we present an unusual case of gastric and duodenal metastases from primary lung SCC with a discussion of the differential diagnosis between metastatic and primary SCC in the stomach and duodenum.

## Case presentation

**Figure 1:**
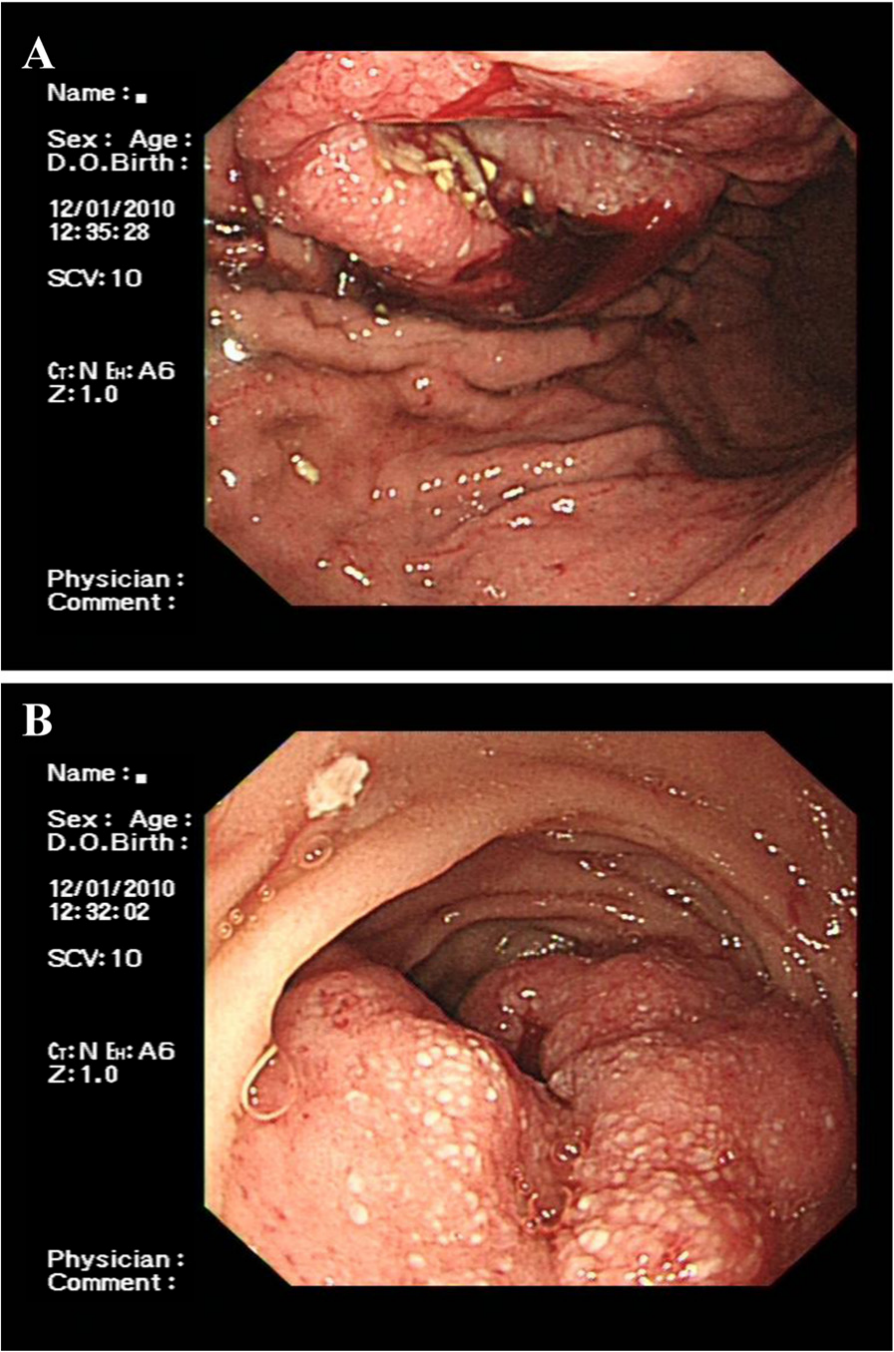
**Gastrointestinal tract metastases revealed by gastroduodenoscopy. (A)** A giant gastric ulcer in the greater curvature of body is shown. **(B)** A huge cauliflower-like mass in the descending part of duodenum is shown.

**Figure 2:**
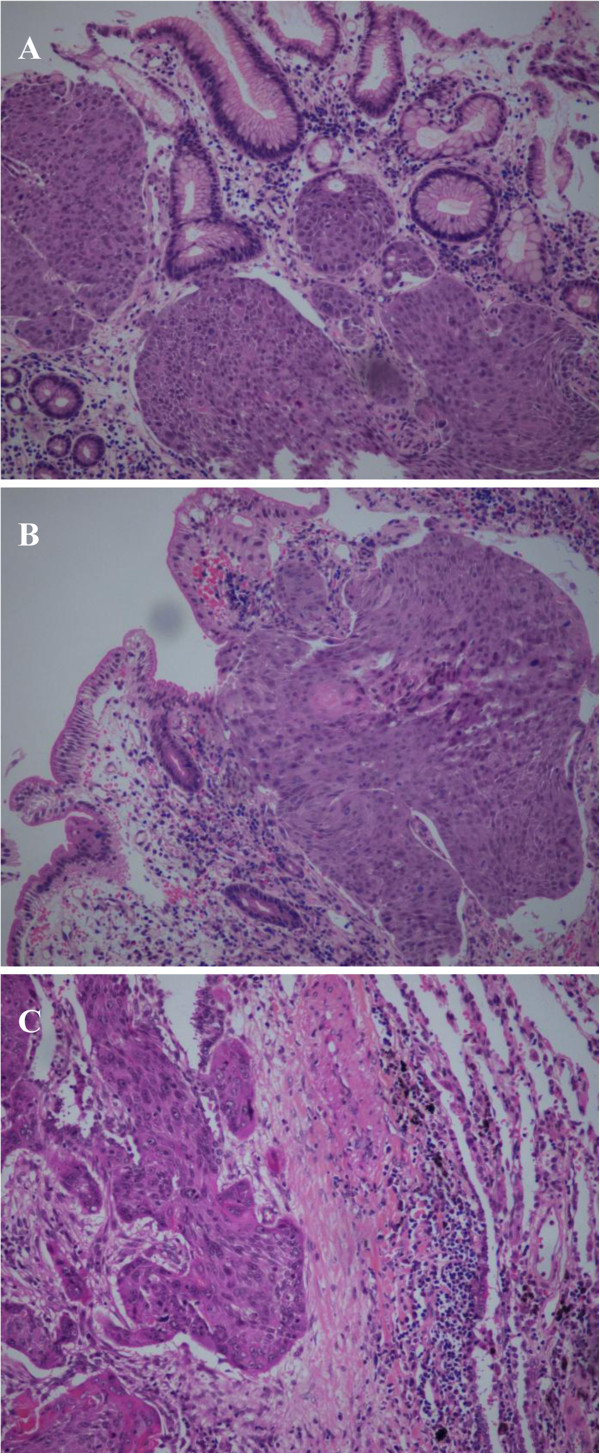
Histopathological views of gastric (A) and duodenal (B) specimens showing squamous cell carcinoma with morphology similar to that of the primary tumor in the lung (C).

**Figure 3:**
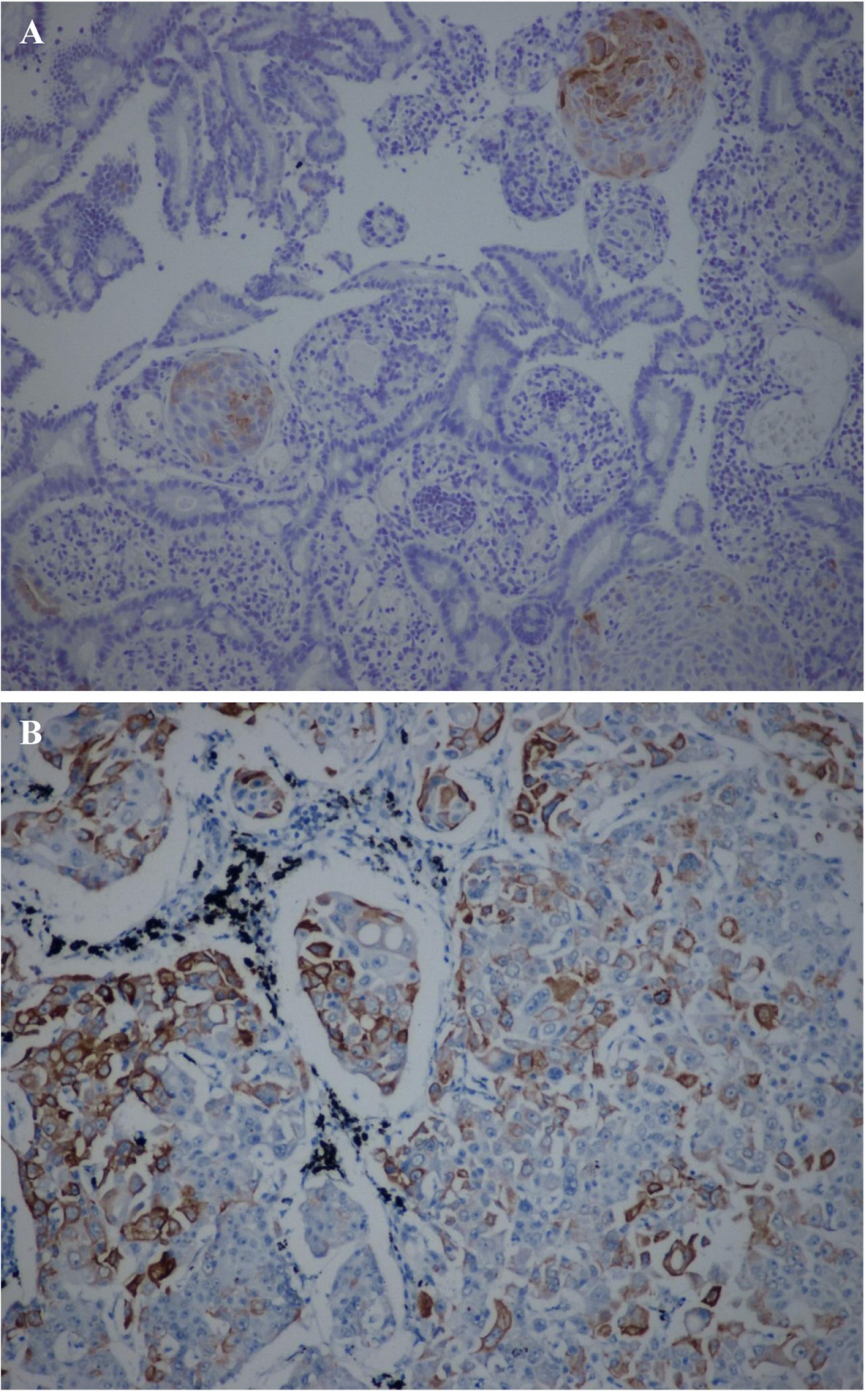
Focal positive staining to cytokeratin 7 of the duodenal lesion (A) and the primary of the lung (B).

A 54-year-old man was referred to our hospital because of a 1-month history of cough. He had smoked one pack of cigarettes per day for the past 20 years. A mass shadow in the hilum of the right lung with an enlarged subcarinal lymph node was found by contrast-enhanced computed tomography (CT) of the chest. A bronchoscopic biopsy yielded the diagnosis of SCC. After metastatic workup with abdominal ultrasonography, magnetic resonance imaging of the brain and bone scan, the patient was staged with T3N1M0 disease. Thereafter he underwent definitive right-middle lobectomy, revealing well- to moderately differentiated SCC and multiple lymph node metastases. He received four cycles of adjuvant chemotherapy comprising taxotere and cisplatin. Five months after his lobectomy, an enlarged mediastinal lymph node was discovered on a routine follow-up chest CT scan. Solitary lymph node recurrence was diagnosed by coincidence circuit single-photon emission CT assessment. The patient then underwent a course of conformal external beam radiation to the mediastinal lymph node at a dosage of 6,000 cGy in 30 fractions. He complained of dysphagia without melena or hematemesis during the fourth week of radiotherapy, which was attributed to radiation esophagitis. However, the patient’s symptoms worsened despite symptomatic treatment. A gastroduodenoscopy performed 10 days after the completion of radiotherapy revealed a giant gastric ulcer in the greater curvature of the stomach with mucosal edema and congestion as well as a huge cauliflower-like mass in the descending part of the duodenum (Figure 1). Biopsies of both lesions revealed SCC. On histopathological examination, hematoxylin and eosin–stained sections of gastric and duodenal biopsies revealed typical SCC and large, eosinophilic cells with distinct cell borders growing beneath the normal gastric and duodenal mucosa (Figure 2). Keratinization, formation of small horn pearls and malignant squamous cells in capillaries were also observed. The morphology of the gastric and duodenal lesions is similar to that of primary tumor of the lung. To confirm the metastatic nature of the lesions, additional immunohistochemical staining analyses of the duodenal lesion were performed. These showed the lesion to stain focally positive for cytokeratin 7 (CK7) and negative for cytokeratin 20 (CK20) and thyroid transcription factor 1 (TTF-1), a staining pattern identical to that of the patient’s primary lung SCC (Figure 3). The patient refused further medical interventions and died of progressive disease 2 months later.

## Discussion

Because of the absence of continuity between the gastric and duodenal metastatic lesions, our present case report is probably the first to describe gastric and duodenal metastases from primary lung SCC. Taking into account the value of identification of metastatic or primary SCC in the stomach and duodenum on the prognosis and treatment options, it is imperative that the correct diagnosis be established. Usually, the visual diagnosis followed by tissue diagnosis of the tumor is enough to establish the diagnosis of SCC. However, it is rarely possible to differentiate metastatic SCC from primary SCC involving the stomach and duodenum based on symptoms and imaging findings, because the major manifestations are the same. A documented history of SCC in other primary sites lends weight to the diagnosis of metastasis, especially in patients with diffuse metastases involving other organs. In this case, recurrence in the mediastinal lymph node and involvement of both stomach and duodenum were highly suggestive of metastatic rather than primary lesions. However, it could be argued that a history of malignancy does not necessarily imply that the new lesions are metastatic in nature. An early-stage primary malignancy followed by new ostensibly metastatic lesions after a relatively long interval will make the differential diagnosis of metastasis versus new primary tumor difficult. More importantly, the diagnosis of metastatic tumor to gastrointestinal tract is challenging on the rare occasions when it represents the first and only metastatic site. It would be even more challenging where the gastrointestinal involvement presents prior to the detection of the primary site.

Histological examination is critical as the most valuable way to differentiate metastatic from primary tumors involving the gastrointestinal tract. The differences in the pathogenensis of metastatic or primary SCC in the gastrointestinal tract are also helpful to the differential diagnosis. The gastrointestinal tract may be metastatically involved by direct invasion, intraperitoneal dissemination and/or lymphatic or hematogenous cancer spread. The pathogenesis of SCC in the stomach and duodenum has not been well-elucidated. Several theories regarding the origin of SCC in the stomach and duodenum have been proposed, including nests of ectopic squamous cells, the proliferation of uncommitted mucosal basal cells into squamous cells, squamous metaplasia secondary to chronic mucosal damage, squamous differentiation in a preexisting adenocarcinonma and multipotent stem cells in the gastrointestinal mucosa [7,10,11]. Endoscopically, nearly all the meatastatic cases present as submucosal tumors with bridging folds and small ulcerations at the top, termed *volcano-like ulcers*[5,12]. The morphologic finding at low magnification of inverted tumor cell growth (that is, with the tumor growth mainly involving the serosal surface and perivisceral adipose tissue with or without ulceration of the mucosa) often suggests a metastatic tumor [13]. Unfortunately, these features can be obscured by biopsy artefact and will disappear as the tumor progresses.

Patients with gastrointestinal metastases are often asymptomatic. Less frequently, these metastases can cause various symptoms such as gastrointestinal perforation, obstruction and/or hemorrhage. These nonspecific symptoms may be misinterpreted as indefinite complaints or as a side effect of treatment. For our patient, his complaint of dysphagia was transiently misdiagnosed as radiation-related esophagitis. Therefore, preemptive endoscopic examinations and careful biopsies are recommended in symptomatic patients. The presence of misplaced squamous cell nests or squamous metaplasia in the mucosa adjacent to the infiltrative tumor growth is highly suggestive of a primary SCC. However, in most of the reported cases of primary gastric SCC, precursor lesions were not demonstrated [8].

Immunohistochemistry may also be useful in reaching the correct diagnosis. Over the past decade, expression levels of CK7, CK20 and TTF-1 have been widely used to distinguish pulmonary from gastrointestinal carcinomas, especially when evaluated as a panel of markers [14]. However, these markers are mainly used to discriminate adenocarcinoma or carcinoid tumors from different sites and have limited value in the differential diagnosis of SCC. Kanthan *et al*. adopted CK5, p63 and p16 as immunohistochemical markers to confirm the diagnosis of SCC of the cervix metastatic to the duodenum [15]. Strikingly, Gevaert *et al*. studied the expression profiles of a series human epithelial cancers and their metastases by microarray technology and concluded that SCCs do not reflect their primary tissue expression profile [16]. Huang *et al*. performed immunohistochemical staining to compare the expression pattern of the epithelial-mesenchymal transition markers between primary SCC of the hypopharynx and a metastatic lesion of the duodenum. Impressively, dissociation of E-cadherin in cellular junctions and nuclear expression of Snail were detected in the primary tumor sample; in contrast, restoration of membranous E-cadherin and disappearance of nuclear Snail expression were demonstrated in the duodenal metastatic sample [17]. Their result may partially explain the unusual metastasis from the metastatic mechanisms, which await further verification.

## Conclusions

The prevalence of SCC of the stomach and/or duodenum is very low. The clinician should be aware of the possibilities of either metastatic or primary involvement of the stomach and duodenum with SCC. Distinguishing between the two requires extensive evaluation, including the patient’s clinical history, histological examination, immunohistochemical staining and possibly microarray data.

## Consent

Written informed consent was obtained from a son of the patient for publication of this case report and any accompanying images. A copy of the written consent is available for review by the Editor-in-Chief of this journal.

## Competing interests

The authors declare that they have no competing interests.

## Authors’ contributions

XS and JH designed the study. JH and YZ drafted the manuscript. All authors contributed to the intellectual content and approved the final version of the manuscript for publication. XS is the guarantor.
